# Impact of Hepatitis B Virus Infection on the Efficacy and Safety of Pembrolizumab plus Chemotherapy for Advanced Biliary Tract Cancer in the KEYNOTE-966 Study

**DOI:** 10.1158/2767-9764.CRC-25-0633

**Published:** 2026-03-17

**Authors:** Stephen L. Chan, Thomas Yau, Robin K. Kelley, Richard S. Finn, Changhoon Yoo, Junji Furuse, Zhenggang Ren, Heinz-Josef Klümpen, Masato Ozaka, Uwe Pelzer, Cagatay Arslan, Joon Oh Park, Julien Edeline, Juan W. Valle, Makoto Ueno, Arndt Vogel, Liis Starkopf, Usha Malhotra, Abby B. Siegel, Shukui Qin

**Affiliations:** 1 State Key Laboratory of Translational Oncology, Department of Clinical Oncology, Sir Y.K. Pao Centre for Cancer, The Chinese University of Hong Kong, Hong Kong, Hong Kong SAR, China.; 2 The University of Hong Kong, Hong Kong, Hong Kong SAR, China.; 3 University of California, San Francisco, San Francisco, California.; 4 University of California, Los Angeles, Los Angeles, California.; 5 Asan Medical Center, University of Ulsan College of Medicine, Seoul, Republic of Korea.; 6 Kyorin University Hospital, Tokyo, Japan.; 7 Zhongshan Hospital Fudan University, Shanghai, China.; 8 University Medical Center, Groningen, the Netherlands.; 9 The Cancer Institute Hospital of the Japanese Foundation for Cancer Research (JFCR), Tokyo, Japan.; 10 Department of Hematology, Oncology and Cancer Immunology, Charite Campus Mitte, Freie Universität Berlin, Humboldt Universität zu Berlin, Berlin Institute of Health, Berlin, Germany.; 11 Izmir Economy University Medical Point Hospital, Karsiyaka, Turkey.; 12 Samsung Medical Center, Sungkyunkwan University School of Medicine, Seoul, Republic of Korea.; 13 Centre Eugène Marquis, Rennes, France.; 14 Cholangiocarcinoma Foundation, Salt Lake City, Utah.; 15 Division of Cancer Sciences, University of Manchester, Manchester, United Kingdom.; 16 Kanagawa Cancer Center, Yokohama, Japan.; 17 Division of Gastroenterology and Hepatology, Medical Oncology, Toronto General Hospital, Princess Margaret Cancer Centre, University of Toronto, Toronto, Canada.; 18 Department of Gastroenterology, Hepatology, Infectious Diseases and Endocrinology, Hannover Medical School, Hannover, Germany.; 19 Merck & Co., Inc., Rahway, New Jersey.; 20 Nanjing Tianyinshan Hospital of China Pharmaceutical University, Nanjing, China.

## Abstract

**Purpose:**

In the randomized phase 3 KEYNOTE-966 trial, first-line pembrolizumab plus chemotherapy significantly improved overall survival (OS) versus placebo plus chemotherapy for participants with advanced biliary tract cancer (BTC). This *post hoc* analysis investigated whether hepatitis B virus (HBV) infection affected the efficacy and safety of pembrolizumab plus chemotherapy.

**Patients and Methods:**

Eligible participants had histologically confirmed extrahepatic or intrahepatic cholangiocarcinoma or gallbladder cancer, unresectable locally advanced or metastatic disease measurable per RECIST v1.1, known HBV and HCV status (including active HBV), and Eastern Cooperative Oncology Group performance status 0 or 1. Participants were randomly assigned 1:1 to intravenous pembrolizumab 200 mg or placebo every 3 weeks for ≤35 cycles plus gemcitabine and cisplatin. HBV-positive participants were monitored for HBV reactivation, and antiviral therapy was required for chronic HBV infection. The primary end point was OS.

**Results:**

The intention-to-treat population comprised 1,069 participants (533, pembrolizumab group; 536, placebo group). The median time from randomization to data cutoff (November 14, 2023) was 36.6 months (range, 29.2–49.4). Among 329 HBV-positive participants (30.8%), 30 had chronic and 299 had clinically resolved HBV. The OS HR was 0.87 [95% confidence interval (CI), 0.69–1.10] with pembrolizumab versus placebo for the HBV-positive subgroup and 0.85 (95% CI, 0.73–0.99) for the HBV-negative subgroup. Safety was consistent between the subgroups. Eight participants had HBV reactivation (5, pembrolizumab group; 3, placebo group), and no cases of HBV-associated hepatitis occurred in either group.

**Conclusions:**

Efficacy and safety outcomes were consistent between HBV-positive and HBV-negative participants receiving first-line pembrolizumab compared with placebo plus gemcitabine and cisplatin.

**Significance:**

Chronic HBV infection is a risk factor for BTC, potentially affecting the effectiveness of BTC treatment. In this study, treatment outcomes in participants who received first-line pembrolizumab plus gemcitabine and cisplatin for advanced BTC treatment were consistent regardless of HBV infection status.

## Introduction

In addition to chronic liver disease and inflammation, infection with hepatitis B or C virus (HBV or HCV) is an established risk factor for the development of hepatocellular carcinoma (1). Chronic HBV infection has further been identified as a risk factor for biliary tract cancer (BTC; refs. 2, 3). Although patients with active HBV or HCV infection are often excluded from clinical trials, a recent review of the literature suggested that HBV infection should not be a contraindication for immune checkpoint inhibitor therapy (4). Although HBV reactivation is a known risk under chemotherapy, the influence of combination with immune checkpoint inhibitor therapy remains unclear. Furthermore, patients infected with HBV are heterogeneous, including those with clinical resolution, reactivation, and active HBV-associated hepatitis (5, 6).

In the randomized phase 3 KEYNOTE-966 trial (NCT04003636), first-line pembrolizumab plus gemcitabine and cisplatin (pembrolizumab group) significantly improved overall survival (OS) versus placebo plus gemcitabine and cisplatin (placebo group), while maintaining health-related quality of life, in patients with advanced BTC [HR, 0.83; 95% confidence interval (CI), 0.72–0.95; *P* = 0.0034; refs. 7, 8]. KEYNOTE-966 provides a prospective database to examine outcomes for patients with HBV infection who received a combination of an immune checkpoint inhibitor and chemotherapy. In this *post hoc* analysis, we determined whether there was an association between HBV infection status and the efficacy and safety of pembrolizumab plus gemcitabine and cisplatin in KEYNOTE-966.

## Patients and Methods

### Study population

The study design and key eligibility criteria of the KEYNOTE-966 study have been previously published. Briefly, eligible participants had histologically confirmed extrahepatic or intrahepatic cholangiocarcinoma or gallbladder cancer, unresectable locally advanced or metastatic disease measurable per RECIST v1.1, no prior systemic therapy, known HBV and HCV status, Eastern Cooperative Oncology Group performance status 0 or 1, and life expectancy >3 months. The study was conducted in accordance with principles of Good Clinical Practice and was approved by the appropriate institutional review boards and regulatory agencies. All participants provided written informed consent.

Participants with clinically resolved HBV [defined as hepatitis B surface antigen (HBsAg) negativity, hepatitis B core antibody (anti-HBc) positivity, and HBV DNA <20 IU/mL] were eligible for enrollment. Participants with chronic HBV infection (defined as HBsAg positivity and/or HBV DNA ≥20 IU/mL) were eligible for enrollment if they had no active HCV infection, started antiviral therapy ≥4 weeks before the start of study treatment, and had HBV DNA <100 IU/mL before the start of study treatment. Participants on active HBV therapy at baseline were monitored for HBV DNA and HBsAg every 12 weeks during study participation and remained on the same HBV therapy throughout study treatment. Participants who were not on active HBV therapy at baseline were monitored for HBV DNA and HBsAg every 6 weeks and started HBV therapy if HBV DNA was ≥100 IU/mL.

In participants with clinically resolved HBV at baseline, HBV reactivation was defined as having HBV DNA >20 IU/mL or reverse HBsAg seroconversion (i.e., reappearance of HBsAg). In participants with chronic HBV at baseline, HBV reactivation was defined as any increase in HBV DNA compared with baseline level or reverse HBsAg seroconversion. In participants with clinically resolved or chronic HBV at baseline, HBV-associated hepatitis was defined as HBV reactivation and hepatitis flare [defined as an alanine aminotransferase (ALT) increase to ≥3× baseline and >100 U/L].

### Procedures and outcomes

Participants were randomly assigned 1:1 to receive intravenous pembrolizumab 200 mg or placebo every 3 weeks for a maximum of 35 cycles plus intravenous gemcitabine 1,000 mg/m^2^ on days 1 and 8 every 3 weeks (no maximum) and intravenous cisplatin 25 mg/m^2^ on days 1 and 8 every 3 weeks (maximum 8 cycles). Participants were stratified by geographic region (Asia vs. not Asia), disease stage (locally advanced vs. metastatic), and site of origin (extrahepatic vs. gallbladder vs. intrahepatic). The primary objective was OS. Secondary objectives were progression-free survival, objective response rate, duration of response assessed per RECIST v1.1 by blinded independent central review, and safety. The primary objective of this analysis was to evaluate OS and safety between treatment groups in participants with or without baseline HBV infection. Incidence of HBV reactivation and HBV-associated hepatitis per the American Association for the Study of Liver Diseases guidelines (6), and use of antiviral medication at any point during the study were additional objectives of interest.

Survival was assessed every 12 weeks until death, withdrawal of consent, or database cutoff date. Adverse events (AE) and laboratory abnormalities were assessed regularly throughout treatment and up to 30 days after discontinuation (through 90 days following cessation of study intervention or 30 days following cessation of study intervention if the participant initiates new anticancer therapy, whichever is earlier, for serious AEs), classified according to the Medical Dictionary for Regulatory Activities (version 25.1), and graded according to the National Cancer Institute Common Terminology Criteria for Adverse Events (version 5). Treatment-related AEs were determined by the investigator to be related to study treatment. Immune-mediated AEs and infusion reactions were based on a list of preferred terms intended to capture known risks of pembrolizumab and were considered regardless of attribution to study treatment by the investigator.

The presence of antibodies against HCV was assessed in blood during screening; hepatitis C viral load was measured if anti-HCV antibodies were present. The presence of antibodies (total and IgM) against HBV core antibody, hepatitis B viral load, and HBsAg were assessed in blood during screening; guidelines for HBV assessment during study treatment are in the protocol.

### Statistical analysis

The current *post hoc* analysis was not powered to assess efficacy. OS was estimated using the Kaplan–Meier method. Efficacy was assessed in all participants randomly assigned to a treatment group (intention-to-treat; ITT population). Safety and treatment exposure were assessed in all randomly assigned participants who received one or more doses of any study treatment (as-treated population). The database cutoff date was November 14, 2023. Data were analyzed using SAS (version 9.4; RRID: SCR_008567). Graphs were redrawn using GraphPad Prism (version 10.6.1; RRID: SCR_002798).

## Results

A total of 1,069 participants comprised the ITT population (533, pembrolizumab group; 536, placebo group). The median time from randomization to database cutoff was 36.6 months (range, 29.2–49.4). Baseline characteristics were generally balanced between treatment groups and HBV infection status (Table 1; Supplementary Table S1). Most participants (*n* = 732; 68.5%) had HBV-negative status at baseline. Among 329 participants (30.8%) with HBV infection at baseline, 30 had chronic HBV infection and 299 had clinically resolved HBV infection. Study participants were representative of the overall population of people diagnosed with BTC in terms of age and sex (Supplementary Table S2).

**Table 1.** Baseline demographics and disease characteristics by HBV infection status (ITT population).

​	HBV-positive *n* = 329		HBV-negative *n* = 732	
	Pembrolizumab + gemcitabine + cisplatin *n* = 164	Placebo + gemcitabine + cisplatin *n* = 165	Pembrolizumab + gemcitabine + cisplatin *n* = 366	Placebo + gemcitabine + cisplatin *n* = 366
Age, median (range), years	66 (39–83)	64 (29–84)	63 (23–85)	62.5 (28–83)
<65	68 (41.5)	86 (52.1)	198 (54.1)	208 (56.8)
≥65	96 (58.5)	79 (47.9)	168 (45.9)	158 (43.2)
Sex	​	​	​	​
Male	102 (62.2)	98 (59.4)	177 (48.4)	170 (46.4)
Female	62 (37.8)	67 (40.6)	189 (51.6)	196 (53.6)
ECOG PS	​	​	​	​
0	61 (37.2)	56 (33.9)	196 (53.6)	168 (45.9)
1	103 (62.8)	109 (66.1)	169 (46.2)	198 (54.1)
≥2	0	0	1 (0.3)	0
Geographic region	​	​	​	​
North America	8 (4.9)	5 (3)	37 (10.1)	33 (9)
Western Europe	11 (6.7)	16 (9.7)	138 (37.7)	134 (36.6)
Rest of the world	145 (88.4)	144 (87.3)	191 (52.2)	199 (54.4)
Disease stage	​	​	​	​
I	1 (0.6)	0	2 (0.5)	2 (0.5)
II	7 (4.3)	5 (3)	12 (3.3)	10 (2.7)
III	9 (5.5)	15 (9.1)	27 (7.4)	31 (8.5)
IV	147 (89.6)	145 (87.9)	325 (88.8)	323 (88.3)
Prior treatment^a^	​	​	​	​
Adjuvant therapy	13 (7.9)	17 (10.3)	34 (9.3)	31 (8.5)
Neoadjuvant therapy	0	0	3 (0.8)	1 (0.3)
Surgery	52 (31.7)	60 (36.4)	106 (29)	101 (27.6)
Radiation	7 (4.3)	9 (5.5)	14 (3.8)	19 (5.2)
Chemotherapy	13 (7.9)	17 (10.3)	37 (10.1)	31 (8.5)
PD-L1 CPS	​	​	​	​
<1	32 (19.5)	37 (22.4)	79 (21.6)	71 (19.4)
≥1	99 (60.4)	92 (55.8)	263 (71.9)	270 (73.8)
Indeterminate	33 (20.1)	36 (21.8)	24 (6.6)	25 (6.8)
MSI status	​	​	​	​
MSI-high	3 (1.8)	1 (0.6)	3 (0.8)	3 (0.8)
Microsatellite stable	119 (72.6)	114 (69.1)	311 (85)	303 (82.8)
Indeterminate	42 (25.6)	50 (30.3)	52 (14.2)	60 (16.4)
Site of origin	​	​	​	​
Gallbladder	26 (15.9)	25 (15.2)	88 (24)	92 (25.1)
Intrahepatic	107 (65.2)	100 (60.6)	211 (57.7)	209 (57.1)
Extrahepatic	31 (18.9)	40 (24.2)	67 (18.3)	65 (17.8)
Hepatitis B status^b^	​	​	​	​
Chronic HBV infection	14 (8.5)	16 (9.7)	0	0
Clinically resolved HBV infection	150 (91.5)	149 (90.3)	0	0
Negative	0	0	366 (100)	366 (100)
Hepatitis C status	​	​	​	​
HCV infection	1 (0.6)	0	0	1 (0.3)
Prior HCV infection	7 (4.3)	9 (5.5)	11 (3)	4 (1.1)
Negative	156 (95.1)	156 (94.5)	355 (97)	361 (98.6)

Data are *n* (%) unless otherwise noted.

Abbreviations: CPS, combined positive score; ECOG PS, Eastern Cooperative Oncology Group performance status; MSI, microsatellite instability.

a Participants who received ≥1 prior treatment were counted for each category of treatment received.

b Eight participants had missing baseline HBV infection status (3, pembrolizumab group; 5, placebo group).

**Figure 1.**
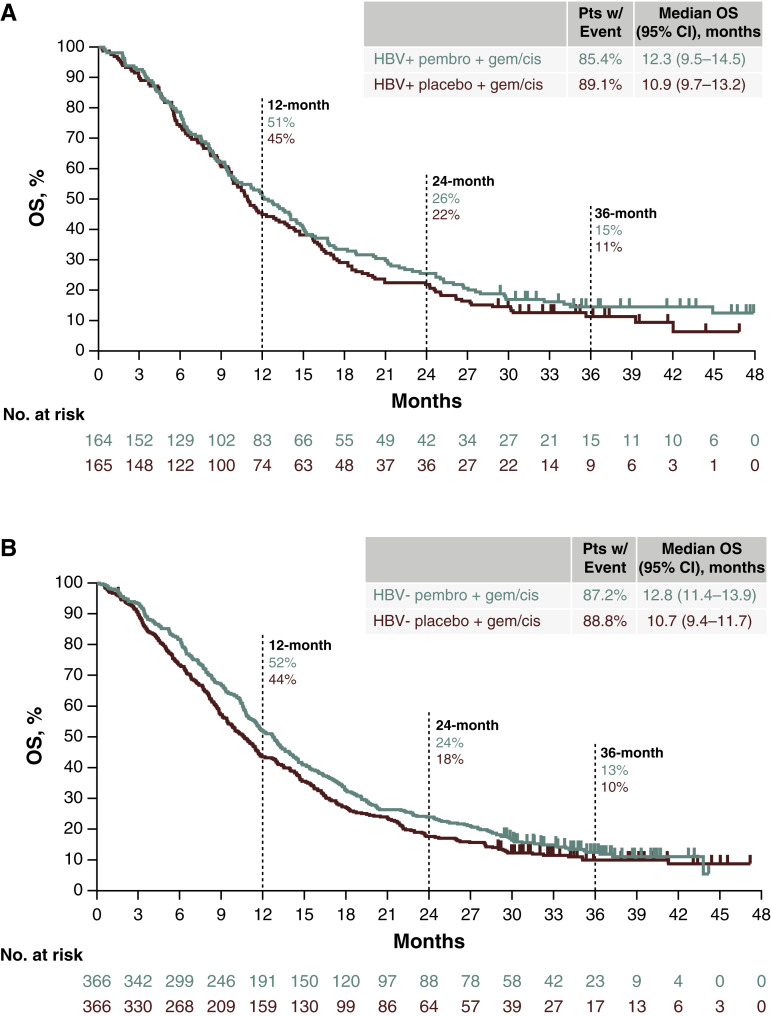
**A,** Kaplan–Meier estimates of OS in participants with HBV infection (ITT population). **B,** Kaplan–Meier estimates of OS in participants without HBV infection (ITT population). Cis, cisplatin; gem, gemcitabine; pembro, pembrolizumab; pts, patients.

**Figure 2.**
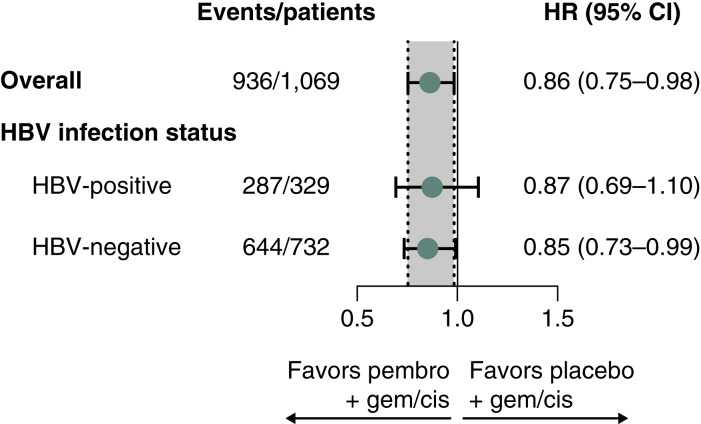
Forest plot analysis of OS by HBV infection status. Cis, cisplatin; gem, gemcitabine; pembro, pembrolizumab.

In the HBV-positive subgroup, 140 participants (85.4%) in the pembrolizumab group and 147 participants (89.1%) in the placebo group had died. The median OS was 12.3 months (95% CI, 9.5–14.5) for the pembrolizumab group and 10.9 months (95% CI, 9.7–13.2) for the placebo group (HR, 0.87; 95% CI, 0.69–1.10; Figs. 1A and 2). In the HBV-negative subgroup, 319 participants (87.2%) in the pembrolizumab group and 325 participants (88.8%) in the placebo group had died. The median OS was 12.8 months (95% CI, 11.4–13.9) for the pembrolizumab group and 10.7 months (95% CI, 9.4–11.7) for the placebo group (HR, 0.85; 95% CI, 0.73–0.99; Figs. 1B and 2). The results were consistent with the ITT population (Fig. 2).

Among the 163 treated participants in the pembrolizumab group with HBV infection at baseline, 9 of 150 (6%) with clinically resolved HBV and all 13 participants (100%) with chronic HBV received antiviral medication during treatment (Supplementary Table S3). Among the 164 treated participants in the placebo group with HBV infection at baseline, 8 of 148 (5.4%) with clinically resolved HBV and 15 of 16 (93.8%) with chronic HBV received antiviral medication during treatment. HBV reactivation occurred in five participants in the pembrolizumab group (4, clinically resolved; 1, chronic) and three participants in the placebo group (1, clinically resolved; 2, chronic; Table 2). For participants who experienced HBV reactivation (HBV DNA >20 IU/mL or reverse HBsAg seroconversion), change in ALT and HBV DNA levels with time, antiviral treatment initiation, and treatment duration were analyzed in participants with clinically resolved HBV infection (Supplementary Fig. S1) and in participants with chronic HBV infection at baseline (Supplementary Fig. S2). No participants in either treatment group had HBV-associated hepatitis.

**Table 2.** HBV-related outcomes in evaluable participants with HBV infection^a^.

​	Pembrolizumab + gemcitabine + cisplatin *n* = 163		Placebo + gemcitabine + cisplatin *n* = 164	
	Clinically resolved *n* = 127	Chronic *n* = 9	Clinically resolved *n* = 128	Chronic *n* = 13
HBV reactivation	4 (3.1)	1 (11.1)	1 (0.8)	2 (15.4)
HBV-associated hepatitis	0	0	0	0
Any HBV DNA increase	4 (3.1)	1 (11.1)	1 (0.8)	2 (15.4)

Data are *n* (%). Percentages were calculated as the number of participants with a given laboratory value divided by the number of participants with baseline and at least one postbaseline laboratory measurement available.

a No participants experienced HBsAg reverse seroconversion or an ALT elevation ≥3× baseline and >100 U/L postbaseline.

In the HBV-positive subgroup, treatment-related AEs occurred in 152 participants (93.3%) in the pembrolizumab group and 156 (95.1%) in the placebo group (Supplementary Table S4). Grade 3 to 5 treatment-related AEs occurred in 113 participants (69.3%) in the pembrolizumab group and 119 (72.6%) in the placebo group. Treatment-related AEs led to death for 1 participant (0.6%) in the placebo group (upper gastrointestinal hemorrhage); no participants in the pembrolizumab group died from treatment-related AEs. Immune-mediated AEs and infusion reactions occurred in 36 participants (22.1%) in the pembrolizumab group and 20 participants (12.2%) in the placebo group.

In the HBV-negative subgroup, treatment-related AEs occurred in 338 participants (93.1%) in the pembrolizumab group and 340 (93.2%) in the placebo group (Supplementary Table S4). Grade 3 to 5 treatment-related AEs occurred in 262 participants (72.2%) in the pembrolizumab group and 249 (68.2%) in the placebo group. Treatment-related AEs led to death for seven participants (1.9%) in the pembrolizumab group (1 each, myocardial infarction; cholangitis; abdominal abscess; lower respiratory tract infection; pneumonia viral; septic shock; and pneumonitis) and two participants (0.5%) in the placebo group (1 each, hepatorenal syndrome and sepsis). Immune-mediated AEs and infusion reactions occurred in 97 participants (26.7%) in the pembrolizumab group and 58 participants (15.9%) in the placebo group.

## Discussion

The results of this exploratory analysis of KEYNOTE-966 suggest that the addition of pembrolizumab to chemotherapy with gemcitabine and cisplatin has comparable efficacy and a similar safety profile regardless of baseline HBV infection status in participants with advanced BTC. There was a low incidence of HBV reactivation and no cases of HBV-associated hepatitis in the HBV-positive subgroup of KEYNOTE-966. With antiviral treatment and careful monitoring of HBV DNA and HBsAg as described, pembrolizumab plus gemcitabine and cisplatin is unlikely to affect underlying HBV in participants with advanced BTC. In addition, patients with clinically resolved HBV infection (anti-HBc–positive) and undetectable HBV DNA are not likely to experience HBV reactivation even without antiviral therapy.

After a median 25.6 months of study follow-up, OS was significantly longer for the pembrolizumab group versus placebo group (HR, 0.83; 95% CI, 0.72–0.95; one-sided *P* = 0.0034) at the final analysis (7). The OS benefit for the pembrolizumab group was maintained after a median follow-up of 36.6 months (HR, 0.86; 95% CI, 0.75–0.98; ref. 9). With an additional 11 months of follow-up, OS numerically favored pembrolizumab in both the HBV-negative (HR, 0.85; 95% CI, 0.73–0.99) and HBV-positive (HR, 0.87; 95% CI, 0.69–1.10) subgroups, although the CI for HBV-positive crossed 1, indicating uncertainty in this subgroup. The apparent difference in treatment effect between HBV-negative and HBV-positive subgroups should be interpreted cautiously. The analysis was not powered for subgroup efficacy, and overlapping Kaplan–Meier curves in the HBV-positive group suggest limited separation early on. This could reflect biological differences in tumor–immune interactions in HBV-related HCC or simply the lack of statistical power due to smaller sample size. Further research is needed to clarify whether HBV status influences response to PD-1 inhibition. However, these data are consistent with those of the randomized phase 3 TOPAZ-1 trial in which first-line durvalumab plus gemcitabine and cisplatin significantly improved OS versus placebo plus gemcitabine and cisplatin in participants with advanced BTC (10, 11). After a median of 41.3 months of follow-up, the HR for OS was 0.74 (95% CI, 0.63–0.87; ref. 11). In addition, 150 of 685 participants (21.9%) had HBV and 22 participants (3.2%) had active HBV (10), similar to KEYNOTE-966 (28.2% and 2.8%, respectively). Notably in KEYNOTE-966, more participants with BTC in the HBV-positive subgroup were from Asia, 76.9% versus 31.8% in the HBV-negative subgroup (12).

The present analysis has some limitations. First, most participants were core positive; only 3% had detectable or active viral load. In addition, the screening and monitoring performed in this study was not performed consistently across all regions in clinical practice. Furthermore, these results should be interpreted carefully given that this was an exploratory analysis and was not prespecified in the protocol. For future studies, data and regulatory guidance should support inclusion of participants with HBV when possible (13, 14).

### Conclusions

The OS benefit of pembrolizumab plus gemcitabine and cisplatin versus placebo plus gemcitabine and cisplatin in KEYNOTE-966 was consistent for participants with and without baseline HBV infection. Efficacy and safety outcomes were also comparable between HBV-positive and HBV-negative subgroups. Taken together, these data support pembrolizumab plus gemcitabine and cisplatin as first-line therapy for advanced BTC regardless of HBV infection status, with careful monitoring and antiviral therapy required for participants with active baseline HBV infection.

## Supplementary Material

Figure S1 Figure S1 shows time course of HBV DNAa and ALT levels, duration of treatment, and initiation of new antiviral therapy in participants with clinically resolved HBV at baseline who experienced HBV reactivation during the study

Figure S2 Figure S2 shows time course of HBV DNAa and ALT levels, duration of treatment, and initiation of new antiviral therapy in participants with chronic HBV at baseline who experienced HBV reactivation during the study

Table S1 Baseline demographic and disease characteristics by chronic and clinically resolved HBV infection (ITT population)

Table S2 Summary of antiviral therapy in treated participants with HBV infection who received antiviral medication during the study

Table S3 Summary of adverse events by HBV infection status (APaT population)

Table S4 Representativeness of study participants.

## Data Availability

Merck Sharp & Dohme (MSD) LLC, a subsidiary of Merck & Co., Inc., is committed to providing qualified scientific researchers access to anonymized data and clinical study reports from the company’s clinical trials for the purpose of conducting legitimate scientific research. MSD is also obligated to protect the rights and privacy of trial patients and, as such, has a procedure in place for evaluating and fulfilling requests for sharing company clinical trial data with qualified external scientific researchers. The MSD data-sharing website (available at https://externaldatasharing-msd.com/) outlines the process and requirements for submitting a data request. Applications will be promptly assessed for completeness and policy compliance. Feasible requests will be reviewed by a committee of MSD subject matter experts to assess the scientific validity of the request and the qualifications of the requestors. In line with data privacy legislation, submitters of approved requests must enter into a standard data-sharing agreement with MSD before data access is granted. Data will be made available for request after product approval in the United States and European Union or after product development is discontinued. There are circumstances that may prevent MSD from sharing requested data, including country- or region-specific regulations. If the request is declined, it will be communicated to the investigator. Access to genetic or exploratory biomarker data requires a detailed, hypothesis-driven statistical analysis plan that is collaboratively developed by the requestor and MSD subject matter experts; after approval of the statistical analysis plan and execution of a data-sharing agreement, MSD will either perform the proposed analyses and share the results with the requestor or will construct biomarker covariates and add them to a file with clinical data that is uploaded to an analysis portal so that the requestor can perform the proposed analyses.
