# Supplementary material for: Impact of Hepatitis B Virus Infection on the Efficacy and Safety of Pembrolizumab plus Chemotherapy for Advanced Biliary Tract Cancer in the KEYNOTE-966 Study
Source: Cancer Res Commun. 2026 Mar 17;6(3):577–84. doi: 10.1158/2767-9764.CRC-25-0633 (PMC13012029; doi:10.1158/2767-9764.CRC-25-0633)
Supplement: Table S1 — Baseline demographic and disease characteristics by chronic and clinically resolved HBV infection (ITT population) [file crc-25-0633_tablest1.docx]

**Table S1. Baseline demographic and disease characteristics by chronic and clinically resolved HBV infection (ITT population)**

|  | **Chronic HBV Infection**  **n = 30** | | **Clinically Resolved HBV Infection**  **n = 299** | |
| --- | --- | --- | --- | --- |
|  | **Pembrolizumab + Gemcitabine + Cisplatin**  **n = 14** | **Placebo + Gemcitabine + Cisplatin**  **n = 16** | **Pembrolizumab + Gemcitabine + Cisplatin**  **n = 150** | **Placebo + Gemcitabine + Cisplatin**  **n = 149** |
| **Age, median (range), years** | 57.5 (41-79) | 57.0 (29-74) | 66.0 (39-83) | 64.0 (32-84) |
| <65 | 9 (64.3) | 10 (62.5) | 59 (39.3) | 76 (51.0) |
| ≥65 | 5 (35.7) | 6 (37.5) | 91 (60.7) | 73 (49.0) |
| **Sex** |  |  |  |  |
| Male | 10 (71.4) | 11 (68.8) | 92 (61.3) | 87 (58.4) |
| Female | 4 (28.6) | 5 (31.3) | 58 (38.7) | 62 (41.6) |
| **ECOG PS** |  |  |  |  |
| 0 | 9 (64.3) | 8 (50.0) | 52 (34.7) | 48 (32.2) |
| 1 | 5 (35.7) | 8 (50.0) | 98 (65.3) | 101 (67.8) |
| **Geographic region** |  |  |  |  |
| North America | 0 | 0 | 8 (5.3) | 5 (3.4) |
| Western Europe | 2 (14.3) | 3 (18.8) | 9 (6.0) | 13 (8.7) |
| Rest of the world | 12 (85.7) | 13 (81.3) | 133 (88.7) | 131 (87.9) |
| **Disease stage** |  |  |  |  |
| I | 0 | 0 | 1 (0.7) | 0 |
| II | 1 (7.1) | 0 | 6 (4.0) | 5 (3.4) |
| III | 0 | 2 (12.5) | 9 (6.0) | 13 (8.7) |
| IV | 13 (92.9) | 14 (87.5) | 134 (89.3) | 131 (87.9) |
| **Prior treatment^a^** |  |  |  |  |
| Adjuvant therapy | 1 (7.1) | 1 (6.3) | 12 (8.0) | 16 (10.7) |
| Surgery | 8 (57.1) | 5 (31.3) | 44 (29.3) | 55 (36.9) |
| Radiation | 1 (7.1) | 1 (6.3) | 6 (4.0) | 8 (5.4) |
| Chemotherapy | 1 (7.1) | 1 (6.3) | 12 (8.0) | 16 (10.7) |
| **PD-L1 CPS** |  |  |  |  |
| <1 | 3 (21.4) | 2 (12.5) | 29 (19.3) | 35 (23.5) |
| ≥1 | 6 (42.9) | 7 (43.8) | 93 (62.0) | 85 (57.0) |
| Indeterminate | 5 (37.5) | 7 (43.8) | 28 (18.7) | 29 (19.5) |
| **MSI status** |  |  |  |  |
| MSI-high | 1 (7.1) | 0 | 2 (1.3) | 1 (0.7) |
| Microsatellite stable | 7 (50.0) | 9 (56.3) | 112 (74.7) | 105 (70.5) |
| Indeterminate | 6 (42.9) | 7 (43.8) | 36 (24.0) | 43 (28.9) |
| **Site of origin** |  |  |  |  |
| Gallbladder | 0 | 2 (12.5) | 26 (17.3) | 23 (15.4) |
| Intrahepatic | 13 (92.9) | 14 (87.5) | 94 (62.7) | 86 (57.7) |
| Extrahepatic | 1 (7.1) | 0 | 30 (20.0) | 40 (26.8) |
| **Hepatitis C status** |  |  |  |  |
| HCV infection | 0 | 0 | 1 (0.7) | 0 |
| Prior HCV infection | 0 | 0 | 7 (4.7) | 9 (6.0) |
| Negative | 14 (100) | 16 (100) | 142 (94.7) | 140 (94.0) |

^a^No participants received prior neoadjuvant therapy. MSI, microsatellite instability; PD-L1 CPS, programmed cell death ligand 1 combined positive score.
