# Supplementary material for: Impact of Hepatitis B Virus Infection on the Efficacy and Safety of Pembrolizumab plus Chemotherapy for Advanced Biliary Tract Cancer in the KEYNOTE-966 Study
Source: Cancer Res Commun. 2026 Mar 17;6(3):577–84. doi: 10.1158/2767-9764.CRC-25-0633 (PMC13012029; doi:10.1158/2767-9764.CRC-25-0633)
Supplement: Table S2 — Summary of antiviral therapy in treated participants with HBV infection who received antiviral medication during the study [file crc-25-0633_tablest2.docx]

**Table S2. Summary of antiviral therapy in treated participants with HBV infection who received antiviral medication during the study**

|  | **Pembrolizumab + Gemcitabine + Cisplatin**  **n = 163** | | **Placebo + Gemcitabine + Cisplatin**  **n = 164** | |
| --- | --- | --- | --- | --- |
|  | **Clinically resolved**  **n = 150** | **Chronic**  **n = 13** | **Clinically resolved**  **n = 148** | **Chronic**  **n = 16** |
| **Any antiviral medication** | 9 (6.0) | 13 (100) | 8 (5.4) | 15 (93.8) |
| Adefovir dipivoxil | 0 | 0 | 0 | 2 (12.5) |
| Entecavir | 4 (2.7) | 10 (76.9) | 5 (3.4) | 10 (62.5) |
| Entecavir maleate | 1 (0.7) | 0 | 0 | 0 |
| Lamivudine | 3 (2.0) | 0 | 2 (1.4) | 2 (12.5) |
| Tenofovir | 0 | 1 (7.7) | 1 (0.7) | 2 (12.5) |
| Tenofovir disoproxil fumarate | 1 (0.7) | 2 (15.4) | 0 | 2 (12.5) |

Data are n (%). For each treatment group, the percentage of participants was calculated as the number of participants within each category divided by the total number of participants in each category.
