# Supplementary material for: Impact of Hepatitis B Virus Infection on the Efficacy and Safety of Pembrolizumab plus Chemotherapy for Advanced Biliary Tract Cancer in the KEYNOTE-966 Study
Source: Cancer Res Commun. 2026 Mar 17;6(3):577–84. doi: 10.1158/2767-9764.CRC-25-0633 (PMC13012029; doi:10.1158/2767-9764.CRC-25-0633)
Supplement: Table S3 — Summary of adverse events by HBV infection status (APaT population) [file crc-25-0633_tablest3.docx]

**Table S3. Summary of adverse events by HBV infection status (APaT population)**

|  | **HBV-positive** | | **HBV-negative** | |
| --- | --- | --- | --- | --- |
|  | **Pembrolizumab + Gemcitabine + Cisplatin**  **n = 163** | **Placebo + Gemcitabine + Cisplatin**  **n = 164** | **Pembrolizumab + Gemcitabine + Cisplatin**  **n = 363** | **Placebo + Gemcitabine + Cisplatin**  **n = 365** |
| **Any AE** | 163 (100) | 163 (99.4) | 358 (98.6) | 364 (99.7) |
| Grade 3-5 | 136 (83.4) | 136 (82.9) | 312 (86.0) | 310 (84.9) |
| Serious | 87 (53.4) | 79 (48.2) | 191 (52.6) | 184 (50.4) |
| Death | 11 (6.7) | 13 (7.9) | 19 (5.2) | 37 (10.1) |
| Discontinued ≥1 study medication | 39 (23.9) | 36 (22.0) | 100 (27.5) | 88 (24.1) |
| Discontinued pembrolizumab/placebo | 23 (14.1) | 22 (13.4) | 55 (15.2) | 45 (12.3) |
| Discontinued any chemotherapy | 36 (22.1) | 35 (21.3) | 89 (24.5) | 81 (22.2) |
| Discontinued all study medication | 9 (5.5) | 13 (7.9) | 25 (6.9) | 26 (7.1) |
| **Any treatment-related AE** | 152 (93.3) | 156 (95.1) | 338 (93.1) | 340 (93.2) |
| Grade 3-5 | 113 (69.3) | 119 (72.6) | 262 (72.2) | 249 (68.2) |
| Serious | 30 (18.4) | 29 (17.7) | 93 (25.6) | 55 (15.1) |
| Death | 0 (0) | 1 (0.6) | 7 (1.9) | 2 (0.5) |
| Discontinued ≥1 study medication | 22 (13.5) | 24 (14.6) | 80 (22.0) | 58 (15.9) |
| Discontinued pembrolizumab/placebo | 10 (6.1) | 9 (5.5) | 37 (10.2) | 18 (4.9) |
| Discontinued any chemotherapy | 20 (12.3) | 23 (14.0) | 70 (19.3) | 51 (14.0) |
| Discontinued all study medication | 4 (2.5) | 6 (3.7) | 13 (3.6) | 8 (2.2) |

Data are n (%). APaT, all participants as-treated.
