# Supplementary material for: Impact of Hepatitis B Virus Infection on the Efficacy and Safety of Pembrolizumab plus Chemotherapy for Advanced Biliary Tract Cancer in the KEYNOTE-966 Study
Source: Cancer Res Commun. 2026 Mar 17;6(3):577–84. doi: 10.1158/2767-9764.CRC-25-0633 (PMC13012029; doi:10.1158/2767-9764.CRC-25-0633)
Supplement: Table S4 — Representativeness of study participants. [file crc-25-0633_tablest4.docx]

**Table S4. Representativeness of study participants.**

| **Cancer type** | **Biliary tract cancer** |
| --- | --- |
| **Considerations related to:** | |
| **Sex** | Based on the National Cancer Institute’s Surveillance, Epidemiology, and End Results (SEER) data from 2000 to 2018, biliary tract cancer is diagnosed slightly more frequently in women (54%) than in men (46%) (1). Evidence suggests that female patients with biliary tract cancer have higher overall survival rates than men (2). |
| **Age** | Most biliary tract cancer diagnoses are made in patients are aged ≥40 years (1), with an average age of approximately 70 years in the United States (1, 3). The mortality rate for biliary tract cancer is 5-10 times higher among patients aged ≥75 years (4). |
| **Race/ethnicity** | In KEYNOTE-966, more patients with biliary tract cancer in the HBV-positive subgroup were from Asia, 76.9% versus 31.8 in the HBV-negative subgroup (5). In this study, regardless of regional distribution, baseline demographics were generally balanced between both treatment groups and are reflective of patients with locally advanced unresectable or metastatic biliary tract cancer. |
| **Geography** | Incidence of biliary tract cancer varies widely depending on geographic region, with the most frequent diagnoses in East Asia and South America (4). Regardless of region, biliary tract cancer is often diagnosed at an advanced stage and has a poor prognosis (2, 3). |
| **Other considerations** | Biliary tract cancer arises from the biliary tract epithelium in the background of chronic inflammation (6). Risk factors of biliary tract cancer include liver fluke infection, chronic liver disease, inflammatory biliary tract diseases, obesity, and tobacco and alcohol use (4). Despite differences in risk factors, disease characteristics of biliary tract cancer are generally similar across the global population, with a majority diagnosed at an advanced stage and with a poor prognosis (2, 3). |
| **Overall representativeness of this study** | With a median age of 62.5 to 66 years and almost half of patients aged ≥65 years, the age distribution of our study is similar to the average age distribution of biliary tract cancer reported in the literature (1, 3).  This global study included patients from North America, South America, Europe, Oceana, and Asia, encompassing geographic regions with known higher incidence of biliary tract cancer. The standard of care for these participants, across all regions, was gemcitabine-based combination chemotherapy with a checkpoint inhibitor. |
| HBV, hepatitis B virus; OR, odds ratio | |

1. Jiang Y, Jiang L, Li F, Li Q, Yuan S, Huang S, et al. *BMC Gastroenterol*. 2022;22(1):546.
2. Sever N, Yunusov E, Majidova N, Kocaaslan E, Erel P, Ağyol Y, et al. *J Cancer Res Clin Oncol*. 2025;151:95.
3. Healey MJ, Seal B, Princic N, Black D, Malangone-Monaco E, Azad NS, et al. *Adv Ther*. 2022;39(12):5530-5545.
4. Baria K, De Toni EN, Yu B, Jiang X, Kabadi SM, Malvezzi M. *Gastro Hep Advances*. 2022;1(4):618-626.
5. Yau T, Chan SL, Kelley RK, Finn RS, Yoo C, Furuse J, et al. *J Clin Oncol*. 2024;42:4097.
6. Bridgewater JA, Goodman KA, Kalyan A, Mulcahy MF. Biliary tract cancer: epidemiology, radiotherapy, and molecular profiling. Am Soc Clin Oncol Educ Book. 2016;36:e194-203.
